# Time-series dataset: fondant feeding in overwintering *Apis mellifera* colonies

**DOI:** 10.1186/s13104-026-07711-y

**Published:** 2026-02-13

**Authors:** Igor Kurdin, Aleksandra Kurdina

**Affiliations:** 1 Institute of Information Technology, Warsaw University of Life Sciences, Warsaw, Poland; 2 AmoHive, Warsaw, Poland

**Keywords:** Honey bee, Overwintering, Fondant feeding, Hive microclimate, Temperature/humidity time series, Hive weight, IoT, Non-invasive monitoring

## Abstract

**Objectives:**

Winter mortality of honey bee colonies remains high and reduces early-spring pollination. Fondant feeding is a common emergency intervention, yet quantitative descriptions of colony and microclimate responses—especially homogeneous time-series—are scarce. Milder winters and unstable springs further elevate starvation risk and complicate overwintering. We present a homogeneous time-series dataset that captures the thermal response of overwintering *Apis mellifera* colonies to fondant feeding under these conditions. The dataset fills this gap and supports reproducible protocols for winter and early-spring fondant feeding.

**Data description:**

The dataset covers Ukraine (mild winter, unstable spring; one colony) and Canada (record-warm winter; three colonies; four feeding events). Data were collected non-invasively from identical Internet of Things (IoT) hives equipped with internal/external temperature (°C) and relative humidity (%) sensors, a load-cell scale, and a solar-powered acquisition and transmission unit. Measurements were recorded at fixed intervals to ensure homogeneous time series. The repository provides raw and cleaned data, a variable dictionary, cleaning/imputation rules, an event log, and a validation script, enabling quantitative assessment for forecasting colony development and for designing scheduled fondant feeding to help reduce winter losses and prepare colonies for early-spring pollination.

## Objective

This Data Note provides an open, homogeneous time-series dataset that captures the thermal response of overwintering *Apis mellifera* colonies to fondant feeding under mild winters and unstable springs. The motivation is elevated winter mortality of bees [1] and climate change, which hinder the maintenance of a stable in-hive microclimate [2] and raise the risk of overwintering failure [3]. Emergency fondant feeding is widespread [4], yet datasets enabling quantitative analysis of response dynamics and predictive modeling of colony development remain limited [5]. We used identical IoT hives and recorded internal/external temperature and humidity and hive weight. The dataset spans winter and early-spring feeding episodes in two locations and two seasons, and supports comparisons between colonies. It enables computation of response intensity and duration as non-invasive time-series features that may be explored in secondary analyses of colony dynamics; these features are not proposed as validated biological biomarkers and require independent validation for any biological interpretation. The resource also covers two practical scenarios: reducing winter mortality during mild winters and unstable early-spring conditions; and planned strengthening of colonies to improve early-spring crop pollination efficiency. The dataset is part of a long-term IoT initiative with AmoHive hives [6]; a technical description of the equipment is available elsewhere, alongside analyses of honey robbing as a colony-threatening pattern [7].

## Data description

Data came from two overwintering monitoring campaigns: Kyiv region, Ukraine (≈ 50.45° N, 30.52° E; 2018–2019), and Toronto region, Canada (≈ 43.39° N, 79.23° W; 2020). One colony in Ukraine received a single fondant feeding during a mild winter period, whereas three colonies in Canada received fondant during four synchronized feeding occasions (February–April 2020) under unstable early-spring conditions following an anomalously warm January and a warm February ( ≈ + 7 °C and ≈ + 3 °C above long-term averages). All AmoHive Langstroth hives were made of extruded polystyrene (XPS).

Fondant feeding protocol: Fondant feeding events were routine overwintering management interventions documented in the event log (product brand, hive ID, local date/time with time zone, and fondant mass). In Ukraine, one feeding event was performed (hive 003; 31 December 2018, local midday): ~0.69 kg of commercial fondant was placed directly on the top bars above the winter cluster in a perforated wrapping, with the perforated side facing downward to allow access. In Canada, fondant feeding occurred during four synchronized feeding occasions: during each occasion, all three colonies (hives 465, 470, and 750) were fed within the same daytime apiary visit using the same placement method. Feeding occasions were scheduled to coincide with short natural warm spells during overwintering, as reflected in the external temperature series (typically > ~ 10 °C at event times). Across the Canadian events, fondant mass ranged from ~ 0.76 to ~ 3.87 kg per feeding event (recorded in the event log, events_all_hives.csv).

Sensors and placement: Internal temperature and humidity were measured using a stationary single-point sensor mounted on the inner rear wall at the level of the 4th frame in the brood box (≈ 5 cm below the top edge). The sensor position is fixed and provides an integrated in-hive microclimate measurement influenced primarily by colony heat production and ventilation-driven air exchange. External temperature and humidity were measured on the rear wall near the lower ventilation opening (≈ 5 cm above the bottom edge). Hive weight was measured by a load-cell platform beneath the hive center.

Hardware and duty cycle: Acquisition used an ATmega328P; transmission a SIMCom SIM5320E with GPS; power from a solar panel and buffer battery. The duty cycle was ~ 95% sleep and ~ 5% active, providing near-homogeneous sampling and power conservation.

Channels and sampling: Primary channels: internal temperature (temp1)/humidity (humid1), external temperature (temp2)/humidity (humid2), and hive weight (weight). The median sampling step was ≈ 1 h. Raw-series completeness by hive ranged from ~ 64% to ~ 88%. A more detailed month-by-month summary of external temperature conditions derived from the cleaned time series is provided in the dataset README to support environmental context and cross-study comparability.

Data processing: Cleaned datasets provide a complete time grid. Imputation for temperature and humidity ranged from ~ 0.3% to ~ 14.2% by channel and hive. Cleaning removed duplicates, physically impossible values (e.g., RH > 100% or spurious 0 °C readings), and isolated weight spikes. Gaps caused by nighttime low battery and intermittent connectivity were handled using a hybrid approach, with different methods for short and long gaps.

Imputation decision framework: To support reuse and uncertainty analysis, the imputation logic is summarized as follows:

- Gap-length thresholds and methods: ≤5 h: time-based linear interpolation for temperature, humidity, and weight; ≥6 h: regression-based imputation with an anchoring offset and diurnal/seasonal components.
- Artifact handling rules: Humidity: sustained RH ≥ 100% blocks were treated as artifacts and handled under dedicated rules; External temperature (temp2): prolonged zero plateaus were logged and not interpolated.
- Imputation flagging and uncertainty use: Imputed temperature and humidity values are traceable in the cleaned tables via temp*_method and humid*_method fields (NaN = measured/original); users are encouraged to consult the corresponding raw files alongside these flags for uncertainty analysis; detailed rules and thresholds are provided in methods_ua_v1.0.md, methods_ca_v1.6.md, and the README.

Repository contents: The repository includes raw CSV archives (data_raw.zip); cleaned/imputed tables with event log (data_clean_events.zip, events_all_hives.csv); a metadata package (metadata.zip) with regional method descriptions (methods_ua_v1.0.md, methods_ca_v1.6.md) and a data dictionary (data_dictionary_master.xlsx); and a validation script (plot_figures_2.py) that reproduces overview plots with feeding times. A README, licenses, checksums, manifest, and citation file are provided. Table 1 overviews the datasets.

Validation: The event log is synchronized with series timestamps, enabling identification of “before” and “after” windows for feeding events. The validation script (plot_figures_2.py) generates overview plots with time on the x-axis, internal/external temperature on the left y-axis, and hive weight on the right y-axis, and marks feeding event times. This supports checks of temporal alignment and data integrity as well as visual plausibility of event-related patterns. As an example expected output, users may observe a transient elevation in internal temperature (temp1) on the order of a few degrees (≈ 1–10 °C) around feeding event markers in the Canadian examples, followed by gradual decay toward the prevailing trajectory. This is a qualitative plausibility check and does not imply causality.

FAIR compliance: A persistent DOI, a data dictionary, standardized field names, open HTTPS access, CSV (UTF-8)/XLSX/MD formats with SI units and ISO 8601 timestamps, CC BY 4.0 for data and MIT for code, checksums, a manifest, and an event log are provided.

Potential applications (non-validated): Possible analytical applications include, but are not limited to: (i) event-aligned analysis of thermal and humidity responses following fondant feeding; (ii) comparison of response timing and magnitude between colonies and sites; (iii) development and benchmarking of forecasting or anomaly-detection methods for overwintering conditions; and (iv) evaluation of data quality and imputation strategies in long-term IoT monitoring. The dataset does not establish validated biological thresholds or biomarkers of colony health or strength, and any biological interpretation requires independent validation.

**Table 1:** Overview of data files and datasets

Label	Name of data file/data set	File types (file extension)	Data repository and identifier (DOI or accession number)
	(i) Raw data		
Data set 1	data_raw.zip	ZIP (CSV inside)	Zenodo (10.5281/zenodo.18220572) [8]
	(ii) Cleaned and imputed data with event logs		
Data set 2	data_clean_events.zip	ZIP (CSV, XLSX inside)	Zenodo (10.5281/zenodo.18220572) [8]
Data file 1	events_all_hives.csv	CSV	Zenodo (10.5281/zenodo.18220572) [8]
	(iii) Metadata and method documentation		
Data file 2	metadata.zip	ZIP	Zenodo (10.5281/zenodo.18220572) [8]
Data file 3	methods_ua_v1.0.md	MD	Zenodo (10.5281/zenodo.18220572) [8]
Data file 4	methods_ca_v1.6.md	MD	Zenodo (10.5281/zenodo.18220572) [8]
Data file 5	data_dictionary_master.xlsx	XLSX	Zenodo (10.5281/zenodo.18220572) [8]
Data file 6	README.md	MD	Zenodo (10.5281/zenodo.18220572) [8]
	(iv) Code and compliance artifacts		
Data file 7	plot_figures_2.py	PY	Zenodo (10.5281/zenodo.18220572) [8]
Data file 8	LICENSE_DATA.txt	TXT	Zenodo (10.5281/zenodo.18220572) [8]
Data file 9	LICENSE_CODE.txt	TXT	Zenodo (10.5281/zenodo.18220572) [8]
Data file 10	CITATION.cff	CFF	Zenodo (10.5281/zenodo.18220572) [8]
Data file 11	MANIFEST.md	MD	Zenodo (10.5281/zenodo.18220572) [8]
Data file 12	checksums_sha256.txt	TXT	Zenodo (10.5281/zenodo.18220572) [8]
Data file 13	thumb_f_v3.png	PNG	Zenodo (10.5281/zenodo.18220572) [8]

Table 1 lists the files provided; for readability, items are grouped into four functional categories: (i) raw data, (ii) cleaned and imputed data with event logs, (iii) metadata and method documentation, and (iv) code and compliance artifacts

## Limitations

This dataset is designed primarily for method development, time-series analytics, and event-response modeling under overwintering conditions, rather than for population-level inference. The limited number of colonies, seasons, and geographic locations constrains generalization, but the homogeneous data structure and explicitly documented processing protocols support reproducible comparative methodological studies. The dataset covers only two seasons (2018–2019; 2020) in two regions (Kyiv, Toronto)—one colony with a single winter feeding and three colonies with four feedings—so broader geography, a larger number of colonies, and different *Apis mellifera* subspecies are needed for more robust generalization. Nonetheless, to our knowledge, this is the first open dataset of its kind, aimed at reducing winter losses amid increasingly frequent milder winters. Such milder winters cannot be scheduled; our IoT network across several locations mitigates this constraint only partially. Telemetry gaps may arise from nighttime battery depletion, communication failures, and occasional sensor artifacts; cleaning reduces but does not eliminate the associated uncertainty. Imputation of longer gaps (linear for short gaps, non-linear for longer windows) may introduce error, particularly for humidity and weight. Fondant composition and formulations were not systematically compared, which limits transferability to other application schemes, including managed early pollination. Hive construction is another limitation: the dataset represents only AmoHive Langstroth (XPS) hives; other designs (e.g., wooden hives with different thermal properties) are not covered.

## Data Availability

The data described in this Data Note are openly available on Zenodo ( [https://doi.org/10.5281/zenodo.18220572](https:/doi.org/10.5281/zenodo.18220572) ). Details and direct access links are provided in Table 1 and in Reference [8].

## Abbreviations

GPS: Global positioning system
IoT: Internet of things
XPS: Extruded polystyrene
